# Rare Presentation of Kaposi Sarcoma Mimicking Madura Foot: A Case Report

**DOI:** 10.1002/ccr3.71626

**Published:** 2025-12-07

**Authors:** Jeminah Nyeenya Nakatudde, Rockie Kisekka, Solomon Kibudde

**Affiliations:** ^1^ Division of Radiation Oncology Uganda Cancer Institute Kampala Uganda; ^2^ Department of Orthopaedic Surgery St. Stephens Hospital Kampala Uganda; ^3^ Department of Internal Medicine Makerere University Kampala Uganda

**Keywords:** histopathology, Kaposi sarcoma, mycetoma, radiotherapy

## Abstract

Kaposi sarcoma (KS) is a low‐grade angioproliferative tumor primarily associated with HIV but also seen in immunocompetent individuals. Its presentation can mimic infectious diseases such as Madura foot (mycetoma), a chronic granulomatous infection endemic to tropical regions. Accurate diagnosis is critical as management differs significantly between the two. We present a rare case of Kaposi sarcoma in a 40‐year‐old HIV‐negative female initially misdiagnosed as Madura foot. The patient underwent two wide local excisions without improvement. Symptoms persisted until the diagnosis of KS was confirmed, and she was subsequently treated with external beam radiotherapy (EBRT), receiving 45 Gy in 15 fractions, which yielded a good partial clinical and radiological response and significant symptom relief. This case underscores the diagnostic complexities in endemic regions and highlights the importance of distinguishing Kaposi sarcoma from infectious diseases such as Madura foot. It also highlights the role of radiotherapy in managing KS.

## Key Clinical Message

In areas where both Kaposi sarcoma and mycetoma are endemic, distinguishing between them can be difficult especially with nodular, ulcerated lesions. Accurate diagnosis is crucial as the treatment differs. This case highlights the diagnostic challenges, the importance of early histopathological confirmation and radiotherapy's effectiveness in managing patients with refractory, localized lesions.

## 1 Introduction

Kaposi sarcoma (KS) is a vascular tumor composed of proliferating endothelial cells caused by human herpesvirus 8 (HHV‐8) also referred to as Kaposi sarcoma‐associated herpesvirus (KSHV), which is responsible for all variants of the disease [1, 2]. Uganda has one of the highest reported burdens and incidences of Kaposi Sarcoma (KS) globally. According to GLOBOCAN 2022, KS accounts for 11.3% of all cancer diagnoses in Uganda, with the age‐standardized incidence rate (ASR) of KS estimated at 9.5 per 100,000 population, among the highest in Africa [3]. The high incidence is mainly attributed to the HIV epidemic in Uganda, given the strong association between KS and immunosuppression. Although commonly associated with HIV/AIDS, KS can also occur in immunocompetent individuals. It manifests with a wide spectrum of clinical presentations, ranging from indolent skin lesions to aggressive visceral involvement.

Madura foot, or mycetoma, is a chronic, slowly progressive infection of the subcutaneous tissues, skin, and bone caused by certain fungi (eumycetoma) or bacteria (actinomycetoma). It is recognized as a neglected tropical disease by the World Health Organization due to its prevalence among underserved populations in tropical and subtropical regions, particularly among individuals exposed to soil‐borne pathogens through minor trauma such as thorn pricks [4]. The burden of mycetoma in Uganda is still largely unrecognized. A study conducted by Kwizera et al. reviewed 70 years of histopathological records and literature and identified 279 cases, with an estimated incidence of 0.32 per 100,000 persons per decade and a prevalence of 8.32 per 100,000. Most of the cases were caused by fungi, predominantly affecting young adult males, with the foot being the most common site [5]. Clinically, mycetoma is characterized by a triad of subcutaneous swelling, multiple sinuses, and discharge containing grains, which are clusters of the causative organisms. Without early diagnosis and treatment, mycetoma can lead to severe complications, including deformity, disability, amputation, and even death [6, 7].

In regions where both mycetoma and Kaposi sarcoma are prevalent, distinguishing between these two conditions is critical. Diagnostic overlap can occur due to shared clinical features such as nodular swelling and ulceration. However, their management differs significantly, with mycetoma requiring antifungal or antibacterial therapy and surgical intervention, while Kaposi sarcoma responds well to radiotherapy and, antiretroviral therapy in HIV‐positive patients. This case highlights a rare instance where Kaposi sarcoma mimicked Madura foot, leading to diagnostic delays and necessitating a multidisciplinary approach to treatment. By emphasizing the diagnostic challenges and the role of radiotherapy in managing KS, as well as the importance of a thorough diagnostic workup in tropical regions.

## 2 Case Presentation

### 2.1 Case History/Examination

A 40‐year‐old HIV‐negative female presented in June 2022 with a progressively enlarging, painful nodular growth on the plantar aspect of her right foot. Initially diagnosed as a benign corn, she underwent wide local excision in July 2022, but symptoms recurred, leading to a second excision in March 2023. Despite these interventions, the lesion persisted, causing significant discomfort and limited mobility.

### 2.2 Differential Diagnosis and Investigations

**FIGURE 1:**
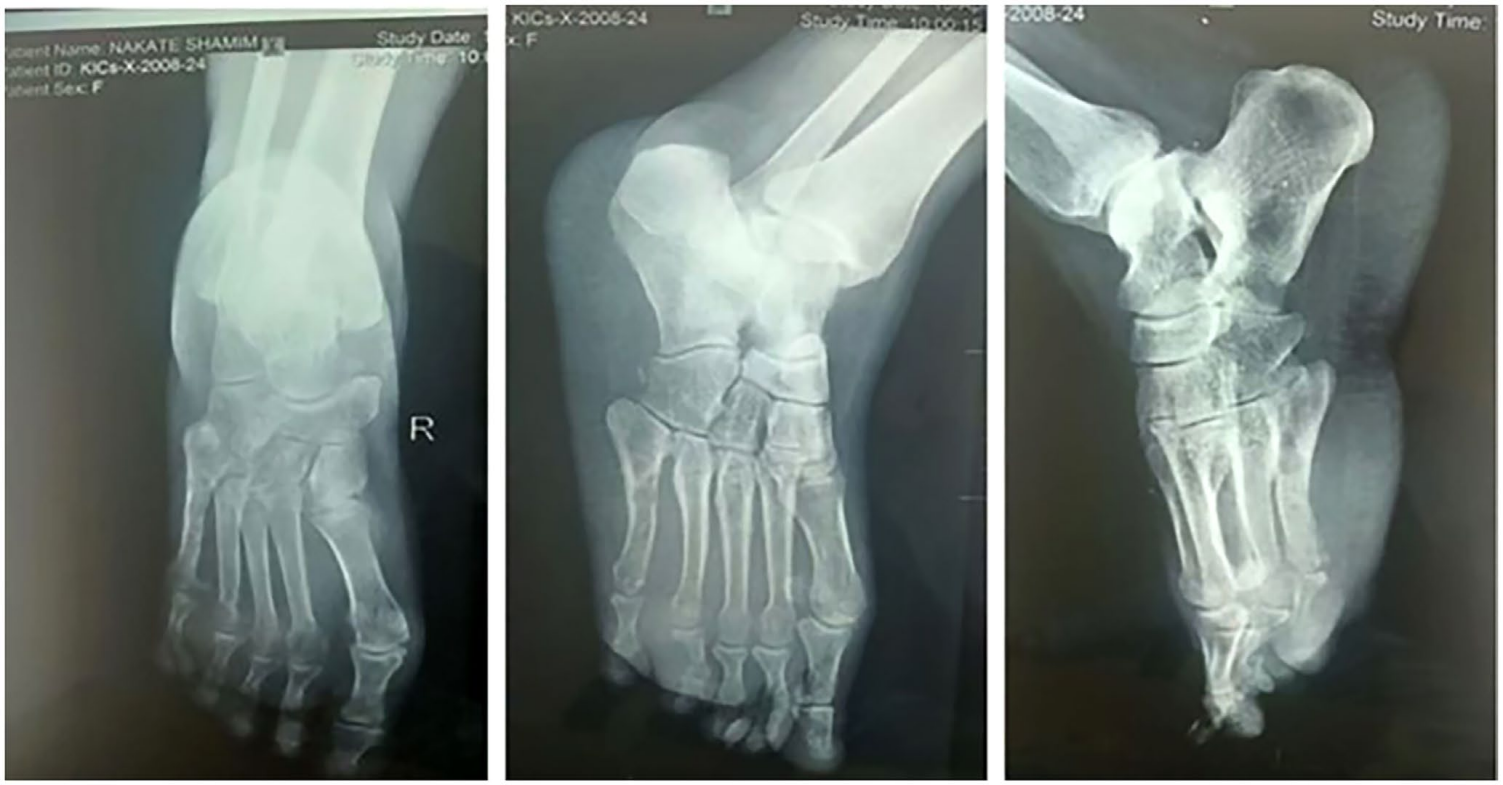
A ‘moth‐eaten’ appearance is observed involving the anterior aspects of the 4th and 5th metatarsals. This is associated with cortical thinning of the bones and marked soft tissue swelling on the plantar aspect of the forefoot.

**FIGURE 2:**
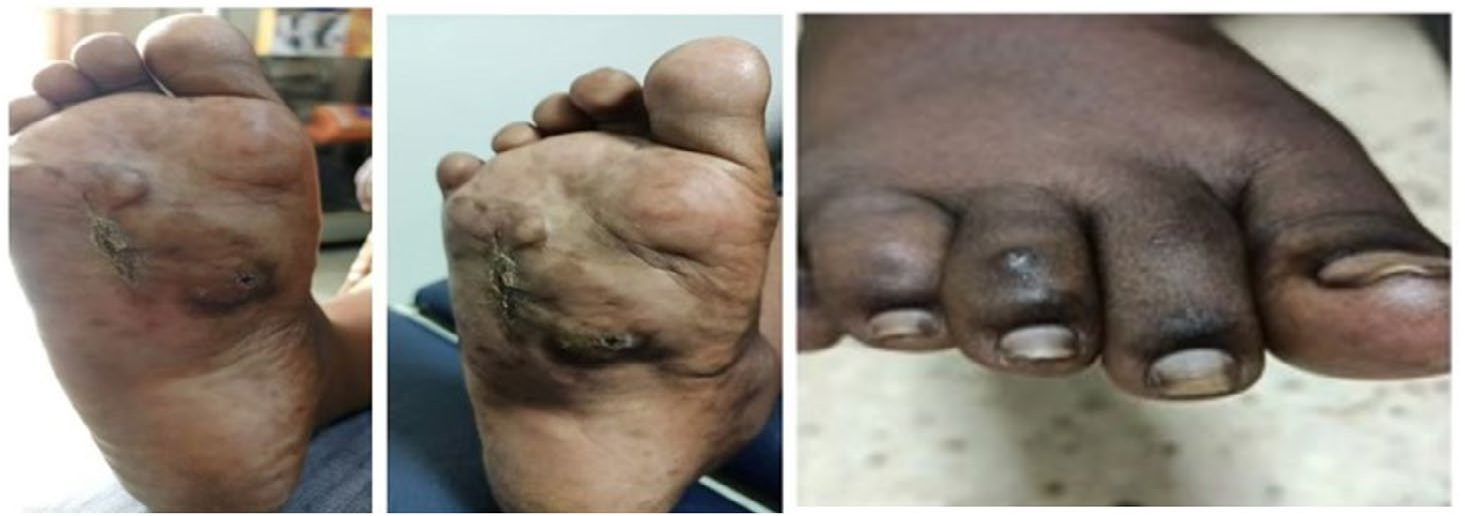
Images of the right foot showing refractory eumycetoma, with a nodular swelling on the plantar aspect measuring 4 × 3 cm.

**FIGURE 3:**
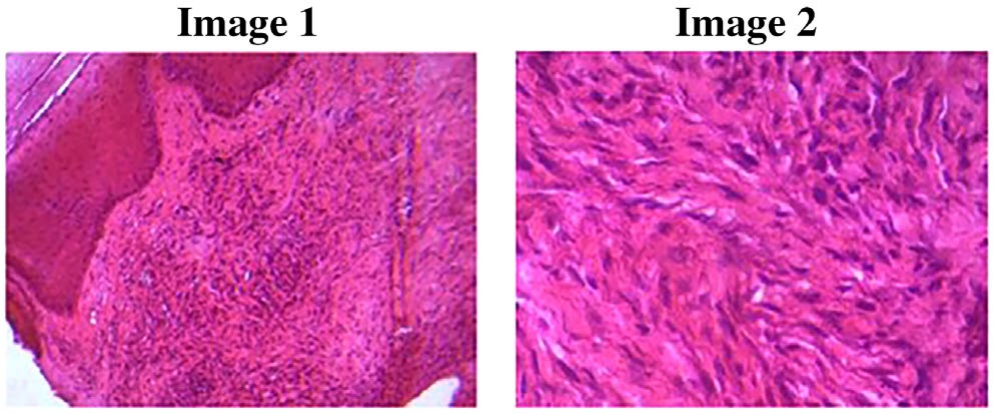
The section shows thick dermal collagen bundles interspersed with slit‐like vascular channels. Extravasation of red blood cells and short fascicles of spindle‐shaped cells are noted, consistent with Nodular Kaposi sarcoma.

In November 2023, imaging revealed an ill‐defined heterogeneous mass measuring 4.67 × 3.56 × 4.34 cm (Figure 1) and a “moth‐eaten” appearance of the fourth and fifth metatarsals on X‐ray (Figure 2). Based on clinical and imaging findings, an initial diagnosis of mycetoma (Madura foot) was made. However, histopathology from an excisional biopsy suggested Kaposi sarcoma at the nodular stage, revealing dermal thick collagen bundles, slit‐like vascular channels, extravasated red cells, and spindle cell fascicles (Figure 3). This confirmed the diagnosis of Kaposi sarcoma, explaining the refractory nature of the lesion. By the time she presented to Uganda Cancer Institute, prior surgeries had failed and amputation had been recommended; however, a limb‐preserving course of external beam radiotherapy (EBRT) was pursued following multidisciplinary review in June 2024.

### 2.3 Treatment, Follow‐Up and Outcome

**FIGURE 4:**
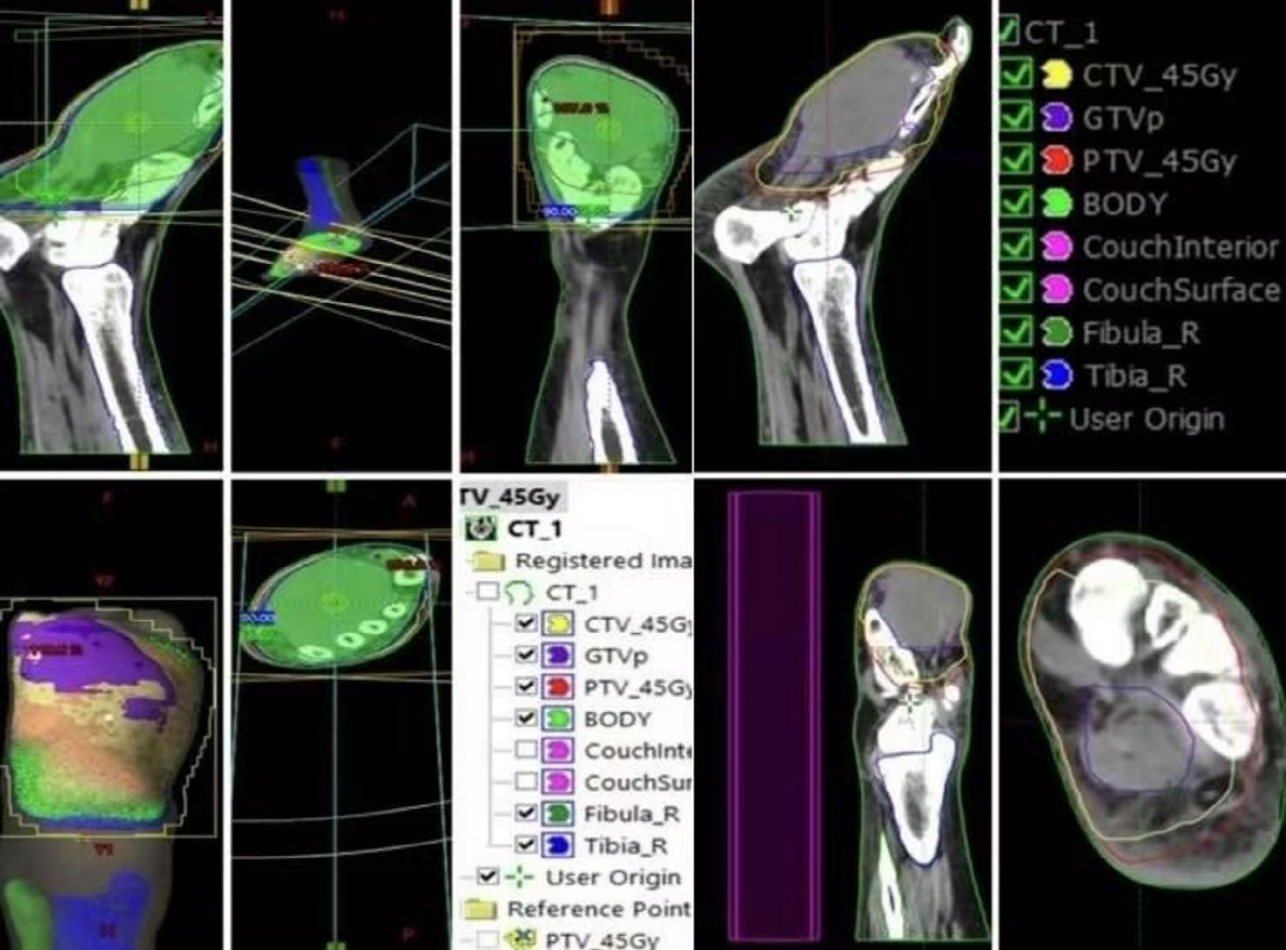
3D Radiotherapy Technique for treatment planning and delivery.

**FIGURE 5:**
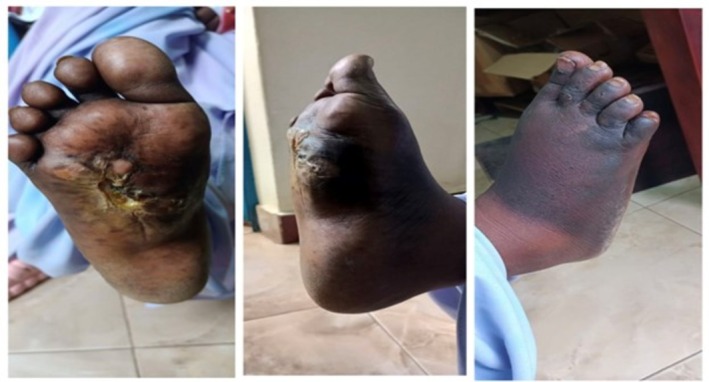
Six weeks post‐EBRT. A 2 cm dry ulcer with surrounding hyperpigmentation is observed, likely a result of EBRT.

The patient received EBRT dose of 45 Gy in 15 fractions, delivered over 3 weeks (Figure 4). At 6 weeks post‐EBRT, a partial response was observed (Figure 5), with a notable reduction in lesion size and resolution of pain. Examination revealed a dry 2‐cm ulcer with hyperpigmentation at the treatment site.

## 3 Discussion

In regions where both infectious and neoplastic conditions are prevalent, ulcerative lesions of the foot often present a diagnostic dilemma [8, 9]. The broad differential diagnosis includes chronic bacterial osteomyelitis, cutaneous tuberculosis, mycetoma chromoblastomycosis and other deep fungal infections. Non‐infectious causes such as squamous cell carcinoma, diabetic or neuropathic ulcers, chronic venous insufficiency and pyoderma gangrenosum may also mimic Kaposi sarcoma both clinically and radiologically. Recognition of this wide spectrum of differentials is essential to avoid delayed or missed diagnosis of KS especially in atypical presentations.

In tropical regions where infectious skin conditions are endemic, the nodular and ulcerative stages of KS can closely resemble chronic granulomatous infections such as Madura foot (Mycetoma). Both may present with progressive nodular swelling, ulceration, and chronic discharging sinuses, which can lead clinicians to favor infection rather than malignancy. This overlap can delay diagnosis as observed in this patient, in whom the working diagnosis remained mycetoma based on clinical appearance supported by initial imaging findings. In endemic regions where Madura foot is prevalent, initial diagnoses often rely on clinical and imaging findings, which may mask neoplastic conditions such as KS.

Endemic KS remains highly prevalent in East and Central Africa, including Uganda which lies within the endemic belt described by Zeinaty et al.(2023). This high regional burden is attributed to widespread HHV‐8 transmission and environmental cofactors that may facilitate viral persistence [10]. In such regions, clinicians should maintain a high index of suspicion for KS even when early findings suggest an infectious process.

Kaposi sarcoma (KS) is a vascular neoplasm with a clinical and histological progression that can resemble other vascular proliferations creating diagnostic uncertainty [11]. It typically progresses from patch to plaque, to nodular stages, and nodular lesions may ulcerate [10]. In endemic settings, these ulcerated lesions reinforce the need for histopathological confirmation rather than clinical judgment alone. In our patient, delayed recognition of this overlap contributed to diagnostic delay and repeated surgical excisions failed to achieve a lasting resolution.

While KS is strongly associated with HIV/AIDS, classical and endemic forms also occur in immunocompetent individuals, although such cases are rare. In this case, the patient was HIV negative and had no comorbidities or secondary causes of immunosuppression making KS clinically less suspected. These atypical host factors necessitated confirmatory imaging and histopathological evaluation.

Diagnosis of KS involves a combination of imaging, biopsy, and histopathological examination, the latter providing a definitive confirmation. Imaging, while valuable in identifying the extent of structural involvement, does not reliably differentiate KS from its mimickers. In this case, imaging revealed a heterogeneous mass with a “moth‐eaten” appearance of the metatarsals (Figure 1), a pattern classically described in mycetoma. However, such radiological features overlap with other pathologies and lack specificity for KS.

In addition to radiological assessment, dermoscopy can serve as a valuable non‐invasive adjunct to improve diagnostic accuracy [12, 13, 14, 15]. The “rainbow pattern” in KS results from polarized light interacting with dermal vascular and fibrotic components, creating multicolored optical dispersion. Although the rainbow pattern was initially described as highly suggestive of KS [13], later studies have shown it is not pathognomonic [12, 14]. Similar optical features may be seen in both vascular and non‐vascular lesions, including aneurysmal dermatofibroma and basal cell carcinoma, and in certain infectious conditions particularly in people of color, where bacterial or fungal infections can mimic KS [12, 14, 15]. Dermoscopy provides useful diagnostic clues, but histopathological confirmation remains the cornerstone of diagnosis, particularly in atypical or resource‐limited settings.

In our setting, dermoscopy was not performed because of limited availability, cost, and the need for operator expertise. In low‐and middle‐income regions, restricted access to equipment and training combined with competing priorities, limits routine dermoscopic evaluation [16]. As a result, clinicians often proceed directly to biopsy with HHV‐8/LANA‐1 immunohistochemistry, which remains the diagnostic gold standard [16, 17, 18]. This highlights the ongoing diagnostic difficulty clinicians often face when differentiating KS from chronic infectious lesions in endemic areas.

Histopathology supported by HHV‐8(LANA‐1) immunohistochemistry is considered the gold standard for confirming KS as nearly all KS lesions are HHV‐8 positive [10]. However, in many resource‐limited settings, access to advanced staining and subspecialty pathology is delayed or unavailable. As reported by Virginie et al. (2003), early KS lesions may be misclassified as benign vascular proliferations such as lymphangioendothelioma, delaying appropriate treatment [18]. Beyond HIV status, clinicians should also consider other contributors to immune dysregulation such as primary immunodeficiencies, diabetes mellitus, or iatrogenic immunosuppression when evaluating atypical KS presentations.

Nodular Kaposi sarcoma (KS) is characterized histologically by fascicles of neoplastic spindle cells and slit‐like vascular channels containing extravasated erythrocytes [11, 19]. In this case, those hallmark features, together with the presence of spindle cells and slit‐like vascular structures on biopsy confirmed KS and explained the lesion's refractoriness to repeated surgical excision. Similar diagnostic dilemmas have been reported in regions where infectious and neoplasia coexist. Verma et al. (2019), described cases where mycetoma was initially mistaken for soft tissue tumors, emphasizing the necessity of a multidisciplinary diagnostic approach in such scenarios [7]. These observations reinforce the need to integrate histopathology and microbiological evaluation into the diagnostic work‐up of destructive foot lesions in endemic areas.

Management of KS is multidisciplinary and may include surgical excision, local therapies, radiation therapy (RT), antiretroviral therapy in HIV‐positive patients, and systemic chemotherapy for disseminated disease.

The Uganda Cancer Institute (UCI) treatment guidelines (2017) recommend tailoring therapy to disease extent and host factors [20]. Moreover, early initiation of antiretroviral therapy (ART) for all HIV‐positive patients is emphasized. Chemotherapy is reserved for advanced or widespread KS, whereas localized radiotherapy typically delivered as external beam radiotherapy (EBRT) is recommended for painful, disfiguring, or recurrent lesions. Fractionated dosing is tailored to the lesion's site and extent. Treatment decisions are guided by ACTG staging, to individualize patient care based on tumor burden, immune status, and systemic illness [20].

For localized KS, local excision, cryotherapy, radiotherapy and intralesional or topical chemotherapy are effective treatment options depending on the lesion size, depth, symptoms, and anatomic site [10, 21]. Radiotherapy is widely used to improve the quality of life with minimal toxicity and is a cornerstone in the management of recurrent, painful lower‐extremity lesions [21, 22]. Multiple fractionation regimens have been reported, and responses may vary based on lesion characteristics and anatomical site [11, 21, 22, 23]. Brenner, et al., reported that radiotherapy achieved an 85% objective response rate in treating Classic KS, with 58% of patients experiencing complete response and 95% reporting symptomatic relief [11]. These outcomes support the use of EBRT with modest fields for local control. For localized lesions, modest doses of radiation applied with a limited margin provide excellent disease control in the treated area. This involves the EBRT delivered once weekly at 4Gy for 6 to 8 consecutive weeks using a 4‐MeV to 6‐MeV electron beam. Treatment ports should encompass the entire skin surface up to 15 cm above the lesion [22].

Radiotherapy played a pivotal role in this patient's management, particularly in addressing bone invasion and recurrent lesions unresponsive to surgery. EBRT, delivered at 45 Gy in 15 fractions, resulted in a partial radiological response and marked symptom relief including pain reduction and improved mobility. These outcomes are consistent with published evidence demonstrating the efficacy of radiotherapy for localized KS, even in immunocompetent individuals. The anti‐inflammatory and antiproliferative effects of radiotherapy further support its use in cases where surgical and pharmacological interventions are insufficient.

This case highlights the need for greater awareness among healthcare providers in endemic, resource‐limited regions. KS should remain a key differential diagnosis for destructive or ulcerative foot lesions, even in HIV‐negative, clinically immunocompetent patients. Early histopathological assessment is essential when presumed mycetoma fails to respond to surgery or antimicrobial therapy. EBRT provides organ‐preserving local control and symptomatic relief when surgery alone is inadequate. Strengthening clinical awareness including among frontline and community health workers to recognize atypical presentations of both infectious and neoplastic conditions may help reduce diagnostic delays and improve outcomes for patients.

## 4 Conclusion

This case underscores the importance of considering Kaposi sarcoma in the differential diagnosis of nodular foot lesions, particularly in regions endemic to mycetoma. Misdiagnosis can lead to delays in appropriate treatment and exacerbate morbidity. Radiotherapy, as demonstrated in this case, remains an effective and well‐tolerated option for localized Kaposi sarcoma. Further research is needed to establish diagnostic protocols and optimize management strategies in resource‐limited settings.

## Author Contributions

**Jeminah Nyeenya Nakatudde:** conceptualization, writing – original draft, writing – review and editing. **Solomon Kibudde:** conceptualization, writing – review and editing. **Rockie Kisekka:** writing – review and editing.

## Funding

The authors have nothing to report.

## Ethics Statement

The authors have nothing to report.

## Consent

Written informed consent was obtained from the patient to publish this report in accordance with the journal's patient consent policy.

## Conflicts of Interest

The authors declare no conflicts of interest.

## Data Availability

No datasets were generated or analyzed during the case report.
